# Individual differences in level of wisdom are associated with brain activation during a moral decision‐making task

**DOI:** 10.1002/brb3.1302

**Published:** 2019-05-01

**Authors:** Michael L. Thomas, Averria S. Martin, Lisa Eyler, Ellen E. Lee, Eduardo Macagno, Mary Devereaux, Winston Chiong, Dilip V. Jeste

**Affiliations:** ^1^ Department of Psychology Colorado State University Fort Collins Colorado; ^2^ Department of Psychiatry University of California San Diego La Jolla California; ^3^ Sam and Rose Stein Institute for Research on Aging University of California San Diego La Jolla California; ^4^ Division of Biological Sciences University of California San Diego La Jolla California; ^5^ Research Ethics Program University of California San Diego La Jolla California; ^6^ Department of Neurology University of California San Francisco San Francisco California; ^7^ Department of Neuroscience University of California San Diego La Jolla California

**Keywords:** brain imaging, compassion, default mode network, insight, neuroscience, psychometrics

## Abstract

**Introduction:**

Wisdom is reportedly associated with better health and quality of life. However, our knowledge of the neurobiology of wisdom is still in the early stages of development. We aimed to improve our understanding by correlating a psychometric measure of the trait with patterns of brain activation produced by a cognitive task theorized to be relevant to wisdom: moral decision‐making. In particular, we aimed to determine whether individual differences in wisdom interact with moral task complexity in relation to brain activation.

**Methods:**

Participants were 39 community‐dwelling men and women aged 27–76 years, who completed moral and nonmoral decision‐making tasks while undergoing functional magnetic resonance imaging. Brain activation in select regions of interest was correlated with participants' scores on the San Diego Wisdom Scale (SD‐WISE).

**Results:**

Individual differences in wisdom were found to interact with brain response to moral versus nonmoral and moral personal versus impersonal dilemmas, particularly in regions in or near the default mode network. Persons with higher scores on the SD‐WISE had less contrast between moral and nonmoral dilemmas and greater contrast between moral‐personal and moral‐impersonal dilemmas than individuals with lower SD‐WISE scores.

**Conclusions:**

Results confirmed our hypothesis that individual differences in level of wisdom would interact with moral condition in relation to brain activation, and may underscore the relevance of considering one's own and others' actions and experiences in the context of wise thinking. Future studies are needed to replicate these findings and to examine specific neurocircuits.

## 1 INTRODUCTION

Discussion about the ubiquitous, and yet ethereal concept of wisdom predates modern psychology and neuroscience (Jeste & Vahia, 2008). Practical wisdom has been considered since the times of Aristotle (Aristotle, 1941) as a means of understanding cognitive, social, and emotional processes involved in decision‐making. In modern day, the fields of psychology, gerontology, and psychiatry have tried to define the concept of wisdom in a way that allows scientific investigation.

Beginning in the 1970s, Baltes, Clayton, and others initiated empirical research on wisdom, focusing on cognitive abilities (Baltes, Smith, & Staudinger, 1992; Clayton & Birren, 1980). Subsequently, several investigators drew attention to the importance of emotional regulation (Ardelt, 2003; Staudinger & Glück, 2011; Sternberg, 1990). Pioneering work by Vaillant, Cloninger, and Blazer stressed the potential role of wisdom in well‐being, health, and longevity (Blazer & Kinghorn, 2015; Cloninger, 2012; Vaillant & Mukamal, 2001).

Numerous studies have now shown that wisdom is associated with positive physical and psychological functioning, including self‐reported physical (Ardelt, 2000), mental health (Ardelt, 2003; Jeste et al., 2013; Roháriková, Špajdel, Cviková, & Jagla, 2013; Thomas, Bangen, Ardelt, & Jeste, 2015; Webster, Westerhof, & Bohlmeijer, 2014), and cognitive functioning (Thomas et al., 2015), among other outcomes. To better leverage these findings into practical mental health applications, more recent efforts have sought to better define wisdom based on its relevant components. Bangen, Meeks, and Jeste (2013) reviewed the literature on wisdom and found six most commonly described components: social advising, emotional regulation, pro‐social behaviors, insight, value relativism, and decisiveness (Bangen et al., 2013; Meeks & Jeste, 2009). The San Diego Wisdom Scale (SD‐WISE) (Thomas et al., 2017) was designed to assess each of the abovementioned six components or domains, and to produce psychometric estimates of the higher‐order wisdom construct.

Meeks and Jeste (2009) postulated a neurobiological basis of wisdom related to the six components defined above. The authors suggested that the prefrontal cortex figures prominently in emotional regulation, social decision‐making, and value relativism via top‐down regulation of the limbic and striatal regions. The dorsolateral prefrontal cortex is presumed to influence calculated, reason‐based decision‐making, whereas the ventromedial prefrontal cortex is implicated in emotional valence and prosocial attitudes and behaviors. Reward neurocircuitry (ventral striatum) is important for promoting prosocial attitudes and behaviors.

This neurobiological basis of individual differences in wisdom is, however, somewhat speculative. We are aware of no prior systematic attempts to correlate measures of wisdom with measures of brain functioning, such as functional magnetic resonance imaging (fMRI). This is due, in part, to the fact that wisdom is difficult to probe using cognitive challenge tasks as is typical in cognitive neuroscience research. An alternative strategy is to correlate psychometric measures of wisdom with patterns of brain activation observed during performance of tasks that require cognitive processes theorized to be related to wisdom.

In particular, moral decision‐making is closely related to wisdom, in that the components of wisdom are assumed to play an important role in its function (Meeks & Jeste, 2009). The association between wisdom and morality dates back, at least, to Aristotle, who believed that wisdom presupposed moral virtuousness (Baltes & Smith, 2008). Indeed, philosophers have argued that wisdom is fundamental to effective decision‐making, as wisdom provides guidance as to what aspects of decisions are truly important (Kupperman, 2005).

Moral decision‐making tasks have been well investigated using fMRI. Greene, Sommerville, Nystrom, Darley, and Cohen (2001) compared brain activation patterns between moral and nonmoral decision‐making conditions (i.e., decisions that involve right/good or wrong/bad behaviors, on the one hand, and decisions where the behaviors are neither moral nor immoral, on the other), and found that moral decisions produced greater activation in brain regions associated with emotional processing (e.g., ventromedial prefrontal cortex and orbital part of the interior frontal cortex) but less activation in brain regions associated with nonemotional processing (e.g., dorsolateral prefrontal cortex). A subsequent body of research has replicated these findings, and also found consistent activation in regions of the bilateral middle temporal cortex, posterior cingulate cortex, precuneus, and caudate nucleus (Garrigan, Adlam, & Langdon, 2017). Similarly, activity in the posterior cingulate cortex and posterior superior temporal sulcus is associated with increased moral sensitivity (Robertson et al., 2007).

When comparing moral personal versus impersonal dilemmas (e.g., those in which the decision‐maker's choice directly violates another person's rights, or not), the personal dilemmas elicit relatively increased activation in what is termed the “default mode network” (DMN; Greene et al., 2001), a functionally connected network of brain regions that typically deactivate during task engagement, and activate during rest or nondirected cognitive activity (Raichle, 2015). The DMN's role in decision‐making may indicate imaginative mental activity related to the dilemma being considered, as well as reflection on one's own experiences (Fossati et al., 2003). Nonpersonal moral dilemmas, on the other hand, elicit relatively greater activity in the cognitive control network (Greene, Nystrom, Engell, Darley, & Cohen, 2004). These findings are particularly relevant to the current study, given that reflective thinking is believed to be a core aspect of wisdom (Ardelt, 2003). It is possible that wise individuals have particularly well‐developed neural mechanisms that support reflective thinking.

To better understand the relationship between wisdom as assessed with a psychometric scale and neurophysiological processes, this study examined whether scores on a psychometric measure of wisdom are associated with patterns of brain activation produced during moral decision‐making. As noted, wisdom is presumed to relate to individual differences in sensitivity to different moral considerations. Consequently, individual differences in wisdom are expected to interact with moral condition in relation to brain activation. This hypothesis is based on two common findings in the neuroimaging literature. First, brain response shows graded effects associated with the demand, or intensity, of motor (Rao et al., 1996), perceptual (Binder et al., 1994; Fox & Raichle, 1984), and cognitive (Callicott et al., 2003; Manoach, 2003) experiments. Second, the demand or intensity of a cognitive experiment is defined relative to the ability of the subject (Brown & Thompson, 2010; Gur, Erwin, & Gur, 1992). That is, it is the difference between the demand of the experiment and the ability of the subject that should determine brain response. Thus, brain activation due to individual differences in sensitivity to different moral considerations—and thus wisdom—is expected to vary with individual differences in psychometrically assessed wisdom. In the moral decision‐making paradigm developed by Greene and colleagues, level of morality engaged by experimental vignettes varies with experimental conditions. We further assumed that wisdom would elicit greater activation within relevant brain regions (e.g., those in the DMN) during moral (vs. nonmoral) and moral‐personal (vs. moral‐impersonal) conditions. Our focus was on several regions of interest (ROIs) previously identified in studies of moral decision‐making (Garrigan et al., 2017), which are mainly, but not exclusively, in the DMN.

## 2 METHODS

### 2.1 Sample

Participants recruited for the current study included 41 community‐dwelling adults who were involved in two ongoing studies: the Successful AGing Evaluation (SAGE) study of community‐dwelling adults (Jeste et al., 2013; Thomas et al., 2016) and a healthy comparison group from a study of schizophrenia (Edmonds et al., 2018; Lee et al., 2018). Cohorts shared the following inclusion and exclusion criteria: (1) community‐dwelling adults, (2) provision of written informed consent to participate in the study, (3) fluency in English, (4) physical and mental abilities to complete the study assessments, (5) no known diagnosis of dementia, and (6) completion of the scale to measure wisdom (SD‐WISE). Additional selection criteria for specific studies are described below.

1. UCSD Successful AGing Evaluation or SAGE cohort (age 21–100 years): This study included community‐dwelling residents of San Diego County who met the following additional inclusion criteria: (a) aged 21–100 years; and (b) had a telephone line within the home. Persons who lived in nursing homes or required daily skilled nursing care, or had a terminal illness were excluded. Participants were recruited using list‐assisted random digit dialing in the San Diego area.
2. Healthy comparison subjects from a study of aging and mental illness (age 26–65 years): These participants were recruited from the greater San Diego area via advertisements for the parent study. Additional exclusion criteria were as follows: (a) past or present major neuropsychiatric illness as screened by the Mini‐International Neuropsychiatric Interview (Sheehan et al., 1998); (b) alcohol or other nontobacco substance abuse or dependence within 3 prior months; and (c) diagnosis of intellectual disability disorder or a major neurological disorder.

Participants invited to the current study were additionally excluded if they had contraindications or conditions incompatible with having an fMRI or had a previous significant head injury. The study protocol was approved by UC San Diego Human Research Subjects Protection Program. All study participants provided a written consent to participate. Data collected for this study are not part of a public data repository.

### 2.2 Measure

All participants were administered the Montreal Cognitive Assessment (MoCA) (Nasreddine et al., 2005) to evaluate global cognitive functioning. The total score ranged from 0 to 30, with higher scores indicating better cognitive performance. The SD‐WISE was administered to assess personal wisdom (Thomas et al., 2017). This scale includes 24 items that are scored using an ordered categorical rating system completed by the examinee. Total scores are taken as the sum of item scores across subscales, taking into account reverse coding as necessary. The total score ranged from 24 to 120, with higher scores indicating higher wisdom.

### 2.3 Moral decision‐making task

Participants completed moral reasoning dilemmas developed in previous research (Chiong et al., 2013; Greene et al., 2004) while undergoing fMRI. Examples of each condition are presented in Table 1. The moral decision‐making task was administered using PsychoPy (Peirce, 2007). Dilemmas were displayed over a series of screens with fixed durations. The first two screens presented the dilemma (17 s each), the third posed a question (5.5 s), and the fourth (6.5 s) was left blank to allow subjects time to respond. There was also a 14 s intertrial interval between dilemmas. Three separate runs of seven dilemmas (7 min each) were presented with an equal number of moral‐personal, moral‐impersonal, and nonmoral conditions per run. For the dilemmas used see (Chiong et al., 2013).

**Table 1:** Examples of nonmoral, moral‐personal, and moral‐impersonal dilemmas from Chiong et al. (2013)

Condition	Screen 1	Screen 2	Screen 3	Utilitarian response
Nonmoral	You are at home one day when the mail arrives. You receive a letter from a company that provides financial services. You have heard of this company, which has a good reputation. They have invited you to invest in a mutual fund. The minimum investment for this fund is $1,000	You already know a lot about this particular mutual fund. It has performed poorly over the past few years. Based on what you know, there is no reason to think that it will perform any better in the future	Would you invest $1,000 in this mutual fund in order to make money?	No
Moral‐Personal	A runaway trolley is heading down the tracks toward five workers, and will kill them if it keeps going. You are on a footbridge over the tracks, in between the approaching trolley and the five workers. Next to you on this footbridge is a stranger who is very large	The only way to save the lives of the five workers is to push this stranger off the bridge and onto the tracks below where his large body will stop the trolley. The stranger will die if you do this, but the five workers will be saved	Would you push the stranger onto the tracks to save the five workers?	Yes
Moral‐Impersonal	You are the night watchman in a hospital. One night, an accident in the building next door makes deadly chemicals enter the hospital's air ducts. If you don't do anything, these fumes will enter a room with three patients in it, and they will all die	The only way to save these three patients from dying is to hit a certain switch. This will keep the fumes out of the room with the three patients in it. Instead, the fumes will enter a room with a single patient in it, and he will die	Would you allow the fumes to enter the room with three patients so that the single patient will live?	No

### 2.4 Image acquisition

Participants were scanned using a General Electric (GE) Discovery MR750 3.0 Tesla whole‐body imaging system and a Nova 32‐channel head coil. All scans were acquired during a single 60‐min session. Anatomical scans were based on a T1‐weighted spoiled gradient echo sequence with fast and prospective motion correction imaging options (repetition time [TR] = 7.4 ms; inversion time [TI] = 1060ms; echo time [TE] = 2.3ms; flip angle = 8°; field of view [FOV] = 25.6 cm; matrix size = 320 × 320; in‐plane resolution = 0.8mm; slice thickness = 0.8mm; slices = 204; slice spacing = 0) acquired parallel to the sagittal plane in an interleaved manner. Functional scans sensitive to the T2‐weighted blood‐oxygen‐level dependent (BOLD) signal were collected using a gradient echo pulse sequence with multiband and echo‐planar imaging options (TR = 800ms; TE = 25ms; flip angle = 52°; FOV = 20.8cm; matrix size = 86 × 86; in‐plane resolution = 2.42mm; slice thickness = 2.4mm; slices = 10 [60 effective]; slice spacing = 0; multiband factor = 6) acquired parallel to the intercommissural (AC‐PC) plane in an interleaved manner. Each scan yielded 544 whole‐brain BOLD images, with the first 12 used for multiband reconstruction. TOPUP scans (both anterior–posterior and posterior–anterior phase encoding) were also acquired to correct for gradient distortion.

### 2.5 Image processing

We used local scripts as well as software from Analysis of Functional NeuroImages (AFNI; Ver. 18.1.14; Cox, 1996) and FMRIB Software Library (FSL; Ver. 5.0.10; Jenkinson, Beckmann, Behrens, Woolrich, & Smith, 2012) to process the structural and functional images. We first used AFNI's segmentation tool to remove non‐brain tissue from structural images. In cases where the automated routines performed less than optimally, adjustments were performed manually. Registration of the anatomical images consisted of using AFNI's Talairach tool to automatically align the images with the ICBM‐452 brain template (Rex, Ma, & Toga, 2003) in Talairach space (Talairach & Tournoux, 1988). Functional images were reconstructed using local scripts. Distortions due to inhomogeneities in the B_0_ magnetic field were corrected using FSL's topup tool (Smith et al., 2004). After correction, all images were visually inspected to ensure that B_0_ distortions—especially in the orbitofrontal cortex and the lateral temporal lobe—were reduced. Scanner artifacts (spikes) were removed using AFNI's despike tool. Next, AFNI's alignment tool was used to co‐register functional images within the time‐series and then align them to the (unregistered) structural images. In all cases, the time‐series was visually inspected to identify an optimal base image. We began with a local Pearson correlation cost function (Saad et al., 2009) and a 12 parameter affine transformation, visually inspected the alignment, and then, if the alignment was not satisfactory based on this visual inspection, re‐aligned using other cost functions until achieving satisfactory results. Using AFNI's quality index tool, the Spearman correlation of each volume with the median volume was used to identify outliers and to create a censor file for the time‐series. The cutoff for censoring time points for each subject was based on the maximum of 0.02 (absolute cutoff) and 3.5 times the median absolute deviation (relative cutoff). The co‐registered functional images were then blurred to an effective full‐width at half maximum of 6 mm smoothness using AFNI's blurring tool.

### 2.6 Regions of interest

We selected ROIs that have been reported to be significantly activated by moral decision‐making tasks, based on results from a quantitative meta‐analysis (Garrigan et al., 2017). ROIs in the frontal lobe included regions mostly comprising the left superior frontal gyrus (SFGmed) near the boundaries of Brodmann areas 8 and 9 (right‐anterior‐inferior [RAI] *x* = 6, *y* = −44, *z* = 40, radius = 4mm), the right SFGmed near the boundaries of Brodmann areas 6, 8, and 9 (*x* = −2, *y* = −44, *z* = 36, radius = 7mm), and the right inferior frontal gyrus (IFGOr) pars orbitalis near the boundaries of Brodmann areas 47 and 11 (RAI *x* = −36, *y* = −28, *z* = −12; radius = 4mm). ROIs in the temporal lobe included regions comprising both the left middle and superior temporal gyri (MTG/STG) near the boundaries of Brodmann areas 39, 19, and 22 (RAI *x* = 44, *y* = 64, *z* = 20; radius = 8mm) and the right MTG/STG also near the boundaries of Brodmann areas 39, 19, and 22 (*x* = −44, *y* = 60, *z* = 24; radius = 7mm). The ROI in the parietal lobe was a region mostly comprising the left (and bilateral) precuneus and posterior cingulate gyrus (PCUN/PCG) mostly near the boundaries of Brodmann areas 7 and 31 (RAI *x* = 2, *y* = 60, *z* = 30; radius = 8mm). Finally, there was one ROI in the basal ganglia, mostly comprising the left caudate nucleus (CAU) (RAI *x* = 12, *y* = −4, *z* = 12; radius = 4mm). (NOTE: Radii reflect the varying size of volumes identified by Garrigan et al. (2017). Two of the ROIs identified by the Garrigan et al. (2017), one in the left MTG and the other in left posterior cingulate gyrus, were omitted due to significant overlap with other ROIs.

### 2.7 Statistical analyses

Analyses of behavioral response data were undertaken to help contextualize the neuroimaging results. Specifically, using generalized linear mixed effects models fitted to data using the lme4 R package (Bates, Maechler, Bolker, & Walker, 2014), we examined the association between utilitarian versus nonutilitarian responses and SD‐WISE scores (Table 1; for coding of utilitarian versus nonutilitarian responses, see Chiong et al., 2013). Utilitarian decisions are those that produce the most good, broadly defined (Driver, 2014). We also examined correlations between age, SD‐WISE scores, and MoCA total scores.

Single subject statistical analyses were based on AFNI's deconvolution tool. Specifically, a general linear model (GLM) was applied to each participant's functional images (omitting censored values). The GLM included explanatory variables representing the behavioral paradigm convolved with a model of the hemodynamic response using a gamma function. The design matrix followed the approach outlined by Chiong et al. (2013). Specifically, we aimed to model the period of time during which participants deliberated about their decision. Therefore, the regressor of interest included the second presentation screen (screen 2) combined with the first 9 s of the question and response screens (i.e., the mean + 1 *SD* reaction time upon presentation of the question). One deliberation regressor was specified for each of the three experimental conditions. An additional explanatory variable modeled the first half of the moral dilemma presentation (screen 1). All other time segments were included as part of the intercept. The model also incorporated covariates accounting for linear, quadratic, cubic, and quartic drift, six motion parameters, and eight physiological noise regressors. To account for physiological motion, respiration and cardiac activity were acquired in parallel with the functional images and converted to sines and cosines of the first and second phase cycles modeling the physiological activity (Glover, Li, & Ress, 2000). These first and second‐order regressors were then added to the convolved design matrix, omitting censored volumes in the time‐series. The GLM was performed on a slice‐by‐slice basis with slices re‐assembled into a 3D map. Thus, the physiological regressors had a differential correction depending on slice to account for the differential effects of physiological motion based on brain location. Regression parameter estimates within voxels were then converted to percent signal change.

In ROI analyses, masks based on the coordinates listed above were used to average parameter estimates over voxels within each region. We then fitted linear mixed effects models with random intercepts and slopes (run and moral condition) to the ROI data. Moral condition was examined using Helmert Coding. Helmert coding compares levels of a variable to the mean of all subsequent levels (Darlington & Hayes, 2017). Orthogonal contrasts are considered the most elegant coding scheme and have good power (Cohen, Cohen, West, & Aiken, 2003; Serlin & Levin, 1985). In the current analysis, the first contrast compared nonmoral to the average of both moral conditions and the second compared moral‐personal to moral‐impersonal conditions. The first contrast is consistent with studies that only broadly compare moral and nonmoral decision‐making (Garrigan et al., 2017), and the second explores differences in conditions that are overtly moral. SD‐WISE scores were standardized and fitted as a quantitative variable with a main effect and interaction terms with both of the moral condition contrasts. Parameters were obtained using the lme4 R package (Bates et al., 2014) with restricted maximum likelihood estimation. In select models, MoCA total scores were added as covariates to determine whether associations between wisdom and brain activation were influenced by cognitive functioning. Due to the limited sample size, combined with the large number of ROIs, inferential tests were focused only on the wisdom by moral condition interaction terms. The Benjamini–Hochberg procedure was applied to *p* values for these effects in order to limit the false discovery rate (FDR) to 5% (Benjamini & Hochberg, 1995).

Whole‐brain voxel‐wise analyses were based on AFNI's program for fitting linear mixed effects models to the whole‐brain data (3dLME). The program failed to converge when fitting models at the level of single run data, and therefore, we focused on the data averaged over runs (and therefore could not include random intercepts and slopes for run). We used AFNI's cluster simulation tool to estimate the probability of false positive clusters of statistically significant voxels. Specifically, we used the auto‐correlation function with two‐sided thresholding, an uncorrected *p* value of 0.001, and a corrected alpha value of 0.05. Voxels were required to cluster together at the faces or edges. Input parameters for the auto‐correlation function were based on the average of participants' residual statistical maps after fitting the GLM.

## 3 RESULTS

### 3.1 Demographic and behavioral data

Of the 41 study participants who were successfully recruited, one asked to the end the fMRI scan early due to discomfort with noise and another participant's data were not usable due to an artifact. Thus, we obtained 39 successful fMRI scans. The participants' ages ranged from 27 to 76 years, with a mean of 39 years (*SD* = 14); 54% identified their gender as male, and 10% identified their ethnicity as Hispanic or Latino. In terms of self‐reported race, 85% identified as White, 10% as more than one race, and 5% as Black or African American. Participants reported a mean of 16 years of education (*SD* = 2; range 9 to 20 years). The mean MoCA total score was 27.3 (*SD* = 2; range 21–30). SD‐WISE scores (*M* = 85.05; *SD* = 10.54; range = 64–107) were not significantly associated with MoCA scores (*p* = 0.900).

The mean response time for the moral decision‐making task (upon presentation of the question) was 6 s (*SD* = 3; range 0.4 s–21.9 s). There were no significant correlations between response time and SD‐WISE scores across conditions (Spearman's *ρ* = 0.011, *p* = 0.867), or specifically within the nonmoral (Spearman's *ρ* = 0.01, *p* = 0.916), moral‐personal (Spearman's *ρ* = −0.04, *p* = 0.468), or moral‐impersonal conditions (Spearman's *ρ* = 0.07, *p* = 0.240).

Overall, participants chose the utilitarian option in 73% of responses. They were more likely to choose utilitarian responses in the nonmoral condition (93%) versus either the moral‐personal condition (57%; *p* < 0.001) or the moral‐impersonal condition (69%; *p* < 0.001). Responses in the moral‐personal condition were also less likely to be utilitarian in comparison to the moral impersonal condition (*p* < 0.001).

In the nonmoral condition, participants with lower SD‐WISE scores (based on a median split [84]) chose the utilitarian option in 88% of responses and participants with higher SD‐WISE scores chose the utilitarian option in 99% of responses; moreover, SD‐WISE scores were significantly associated with utilitarian responding within the nonmoral condition overall (*b* = 3.12, *SE* = 0.82, *p* < 0.001). In the moral‐personal condition, utilitarian responses were chosen 55% of the time by participants with lower SD‐WISE scores and 58% of the time by participants with higher SD‐WISE scores. In the moral‐impersonal condition, utilitarian responses were chosen 66% of the time by participants with lower SD‐WISE scores and 74% of the time by participants with higher SD‐WISE scores. However, SD‐WISE scores were not significantly associated with utilitarian responses made during moral‐personal (*b* = 0.15, *SE* = 0.46, *p* = 0.74) or moral‐impersonal (*b* = 0.19, *SE* = 0.31, *p* = 0.54) conditions.

### 3.2 ROI analyses

**Figure 1:**
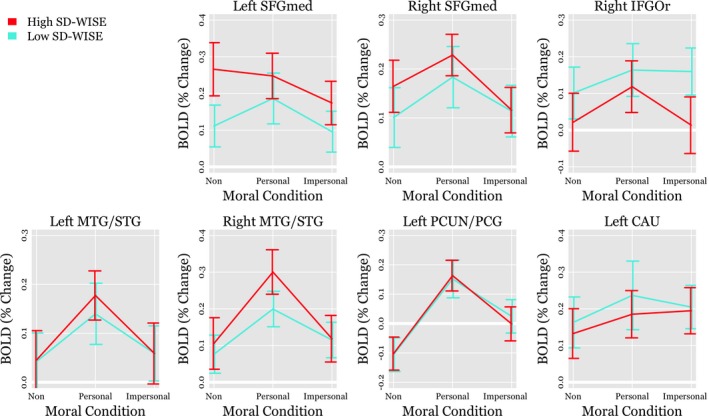
Region of interest (ROI) based means and 95% confidence intervals for the average blood‐oxygenation‐level dependent (BOLD) effect across nonmoral, moral‐personal, and moral‐impersonal conditions. Lines represent two groups of participants with low versus high San Diego Wisdom Scale (SD‐WISE) scores based on a median split. SFGmed, Medial Superior Frontal Gyrus; IFGOr, Inferior Frontal Gyrus Pars Orbitalis; MTG, Middle Temporal Gyrus; PCUN, Precuneus; PCG, Posterior Cingulate Gyrus; CAU, Caudate

Regression parameter estimates for all interaction effects involving SD‐WISE scores within the ROI analyses are reported in Table 2. For interpretive purposes, Figure 1 plots means and 90% confidence intervals for percent signal change in the BOLD response for each ROI, separating participants into those with high versus low SD‐WISE scores based on a median split. After correcting for the FDR, two of the effects were significant: the interactions between SD‐WISE total scores and the moral‐personal versus moral‐impersonal contrast effect in the right IFGOr and MTG/STG. Parameter estimates suggest that participants with higher scores on the SD‐WISE showed a relatively larger difference in activation between moral personal and impersonal conditions in comparison to participants with lower SD‐WISE scores.

**Table 2:** Wisdom interaction effects for region of interest (ROI) analyses

ROI	*x*	*y*	*z*	*R*	SD‐WISE interaction effect	*b*	*SE*	*df*	*t*	*p*_FDR_
Left SFGmed	6	−44	40	3.01	Average of moral minus nonmoral	−0.04	0.02	69.94	−1.78	0.28
Left SFGmed	6	−44	40	3.01	Moral personal minus moral impersonal	0.01	0.03	42.23	0.53	0.93
Right SFGmed	−2	−44	36	7.12	Average of moral minus nonmoral	−0.03	0.02	38.03	−1.30	0.53
Right SFGmed	−2	−44	36	7.12	Moral personal minus moral impersonal	0.05	0.03	69.09	1.95	0.26
Right IFGOr	−36	−28	−12	4.13	Average of moral minus nonmoral	−0.02	0.03	181.97	−0.79	0.79
Right IFGOr	−36	−28	−12	4.13	Moral personal minus moral impersonal	0.09	0.03	120.43	2.82	0.04
Left MTG	44	64	20	7.77	Average of moral minus nonmoral	0.00	0.02	74.11	0.20	0.96
Left MTG	44	64	20	7.77	Moral personal minus moral impersonal	0.03	0.03	149.16	1.22	0.53
Right MTG	−44	60	24	6.68	Average of moral minus nonmoral	−0.01	0.02	85.94	−0.27	0.96
Right MTG	−44	60	24	6.68	Moral personal minus moral impersonal	0.07	0.02	123.78	3.30	0.02
Left PCUN/PCG	2	60	30	7.72	Average of moral minus nonmoral	0.00	0.02	84.76	−0.06	0.96
Left PCUN/PCG	2	60	30	7.72	Moral personal minus moral impersonal	0.02	0.03	42.22	0.76	0.79
Left CAU	12	−4	12	3.98	Average of moral minus nonmoral	0.00	0.03	126.67	0.12	0.96
Left CAU	12	−4	12	3.98	Moral personal minus moral impersonal	−0.01	0.04	106.67	−0.17	0.96

Note

*p*_FDR_ is the *p* value after correcting for a false discovery rate of 5%. *x*, *y*, and *z* are RAI coordinates in Talairach space with radius *R*. Parameter estimates are based on linear‐mixed effects models.

Abbreviations: ROI, region of interest; SFGmed, Medial Superior Frontal Gyrus; IFGOr, Inferior Frontal Gyrus Pars Orbitalis; MTG, Middle Temporal Gyrus; PCUN, Precuneus; PCG, Posterior Cingulate Gyrus; CAU, Caudate.

### 3.3 Whole‐brain voxel‐wise analyses

**Figure 2:**
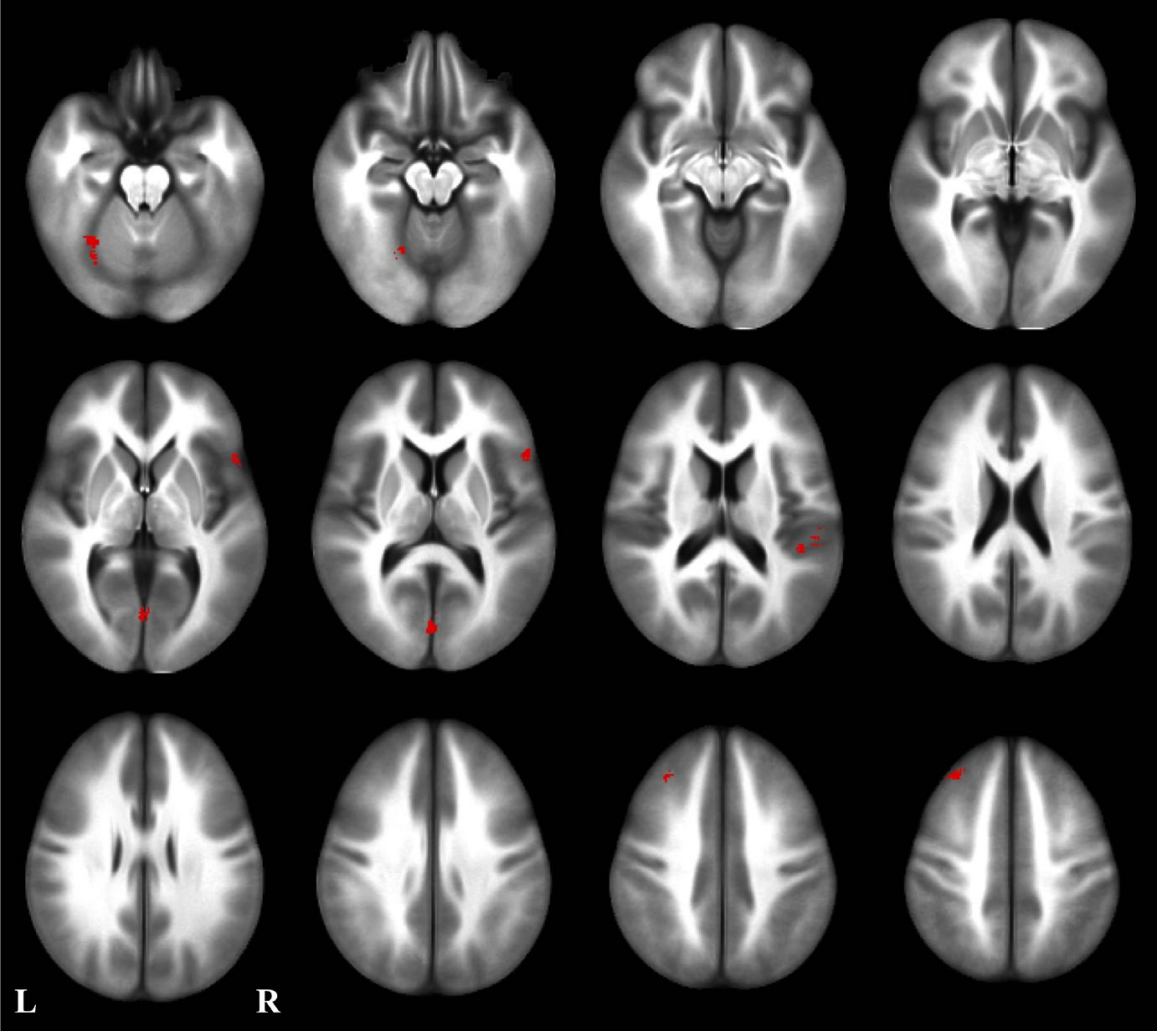
Significant clusters (red) after family wise error corrections of whole‐brain voxel‐wise analysis. Five regions were identified: a cluster in the left dorsal cerebellar cortex, a cluster near calcarine sulcus comprising the more anterior portions of both the cuneus (CUN) and the lingual gyrus (LING), a cluster in the right superior temporal gyrus (STG), a region in the left middle frontal gyrus (MFG), and a region in the right inferior frontal gyrus (IFG)

Whole‐brain voxel‐wise analysis produced five significant clusters after correcting for the family‐wise error rate (see Figure 2): a cluster in the left dorsal cerebellar cortex (CB) (*x* = 28, *y* = −19, *z* = 342, 342 voxels), a cluster near the calcarine sulcus comprising the more anterior portions of both the cuneus (CUN) and the lingual gyrus (LING) near the boundaries of Brodmann areas 18, 17, and 23 (*x* = 2, *y* = 78, *z* = 10, 340 voxels), a cluster in the right STG near the boundaries of Brodmann areas 40, 41, and 42 (*x* = −51, *y* = 22, *z* = 19, 267 voxels), a cluster in the left middle frontal gyrus (MFG) mostly within Brodmann area 8 (*x* = 32, *y* = −33, *z* = 46, 245 voxels), and a cluster in the right inferior frontal gyrus (IFG) mostly within Brodmann area 45 (*x* = −52,* y* = −22, *z* = 8).

**Figure 3:**
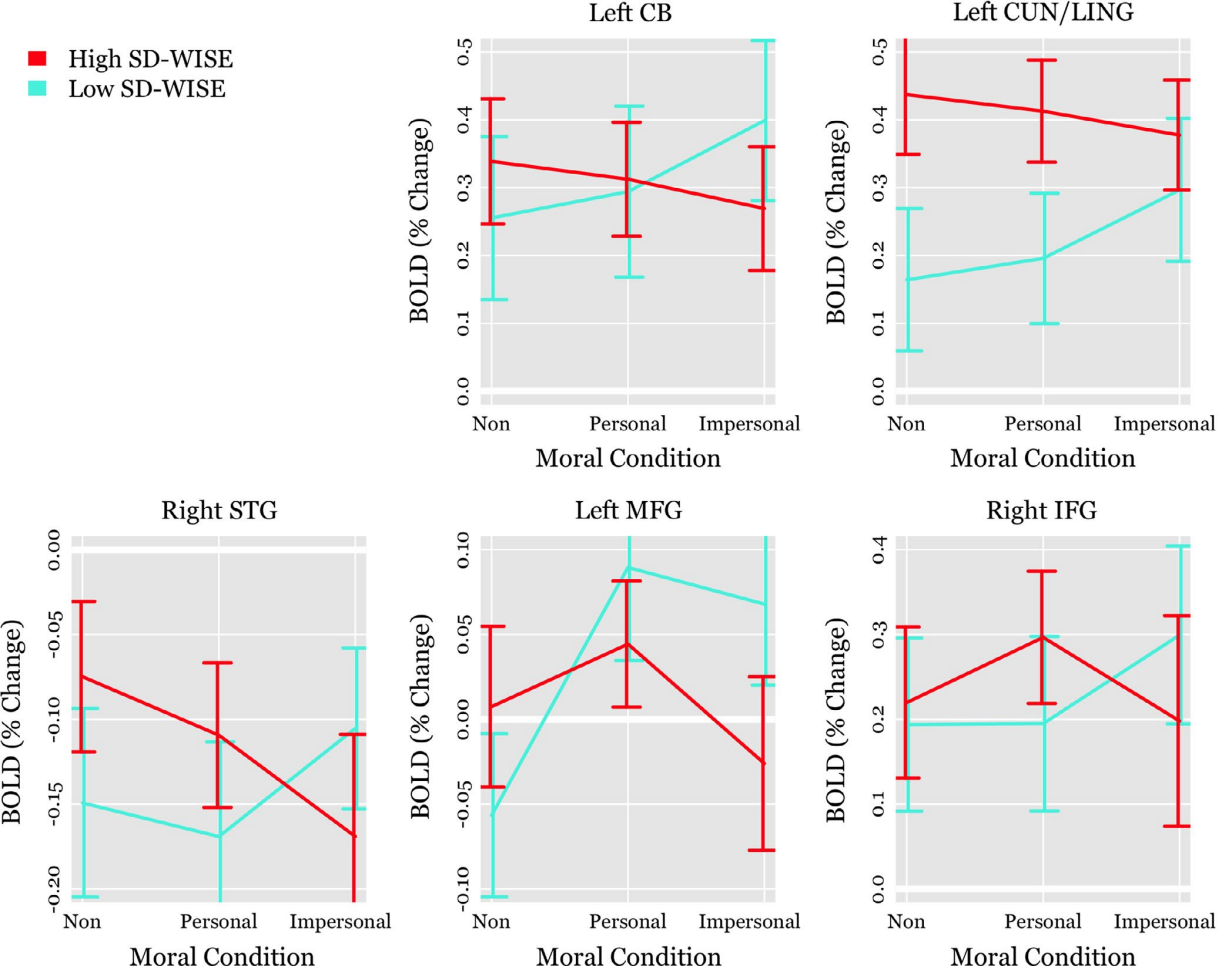
Whole‐brain voxel‐wise analysis based means and 95% confidence intervals for the average blood‐oxygenation‐level dependent (BOLD) effect across nonmoral, moral‐personal, and moral‐impersonal conditions. Lines represent two groups of participants with low versus high San Diego Wisdom Scale (SD‐WISE) scores based on a median split. CB, cerebellum; CUN, cuneus; LING, lingual gyrus; STG, superior temporal gyrus; MFG, middle frontal gyrus

SD‐WISE total scores by moral condition contrast effects using ROIs based on these clusters are reported in Table 3. Corresponding means and confidence intervals are shown in Figure 3. Given that the analyses are based on clusters of voxels already shown to be significantly active in relation to the SD‐WISE total score by moral condition interaction, it is not surprising that all but two of the contrast effect interactions are significant. The regression coefficients show a consistent pattern: participants with higher SD‐WISE total scores demonstrated relatively less percent signal change in the BOLD response during the average of moral versus nonmoral conditions. At the same time, participants with higher SD‐WISE total scores demonstrated relatively more activation in the moral‐personal versus moral‐impersonal condition.

**Table 3:** Wisdom interaction effects for whole‐brain voxel‐wise analyses

ROI	*x*	*y*	*z*	vox	SD‐WISE interaction effect	*b*	*SE*	*df*	*t*	*p*_FDR_
Left CB	28	54	−19	342	Average of moral minus nonmoral	−0.10	0.03	121.50	−3.48	<0.001
Left CB	28	54	−19	342	Moral personal minus moral impersonal	0.10	0.03	145.84	3.05	<0.001
Left CUN/LING	2	78	10	340	Average of moral minus nonmoral	−0.09	0.03	71.15	−2.67	0.01
Left CUN/LING	2	78	10	340	Moral personal minus moral impersonal	0.12	0.04	106.08	3.28	<0.001
Right STG	−51	22	19	267	Average of moral minus nonmoral	−0.06	0.02	187.77	−2.69	0.01
Right STG	−51	22	19	267	Moral personal minus moral impersonal	0.07	0.03	178.15	2.85	0.01
Left MFG	32	−33	46	245	Average of moral minus nonmoral	−0.10	0.02	46.73	−4.30	<0.001
Left MFG	32	−33	46	245	Moral personal minus moral impersonal	0.05	0.02	126.10	2.18	0.03
Right IFG	−52	−22	8	185	Average of moral minus nonmoral	−0.06	0.03	137.18	−1.71	0.09
Right IFG	−52	−22	8	185	Moral personal minus moral impersonal	0.15	0.04	164.23	3.86	<0.001

Note

*x*, *y*, and *z* are RAI coordinates in Talairach space with vox number of voxels within each cluster. Parameter estimates are based on linear‐mixed effects models.

Abbreviations: CB, cerebellum; CUN, cuneus; LING, lingual gyrus; STG, superior temporal gyrus; MFG, middle frontal gyrus; IFG, inferior frontal gyrus.

### 3.4 Standardized effect size maps

**Figure 4:**
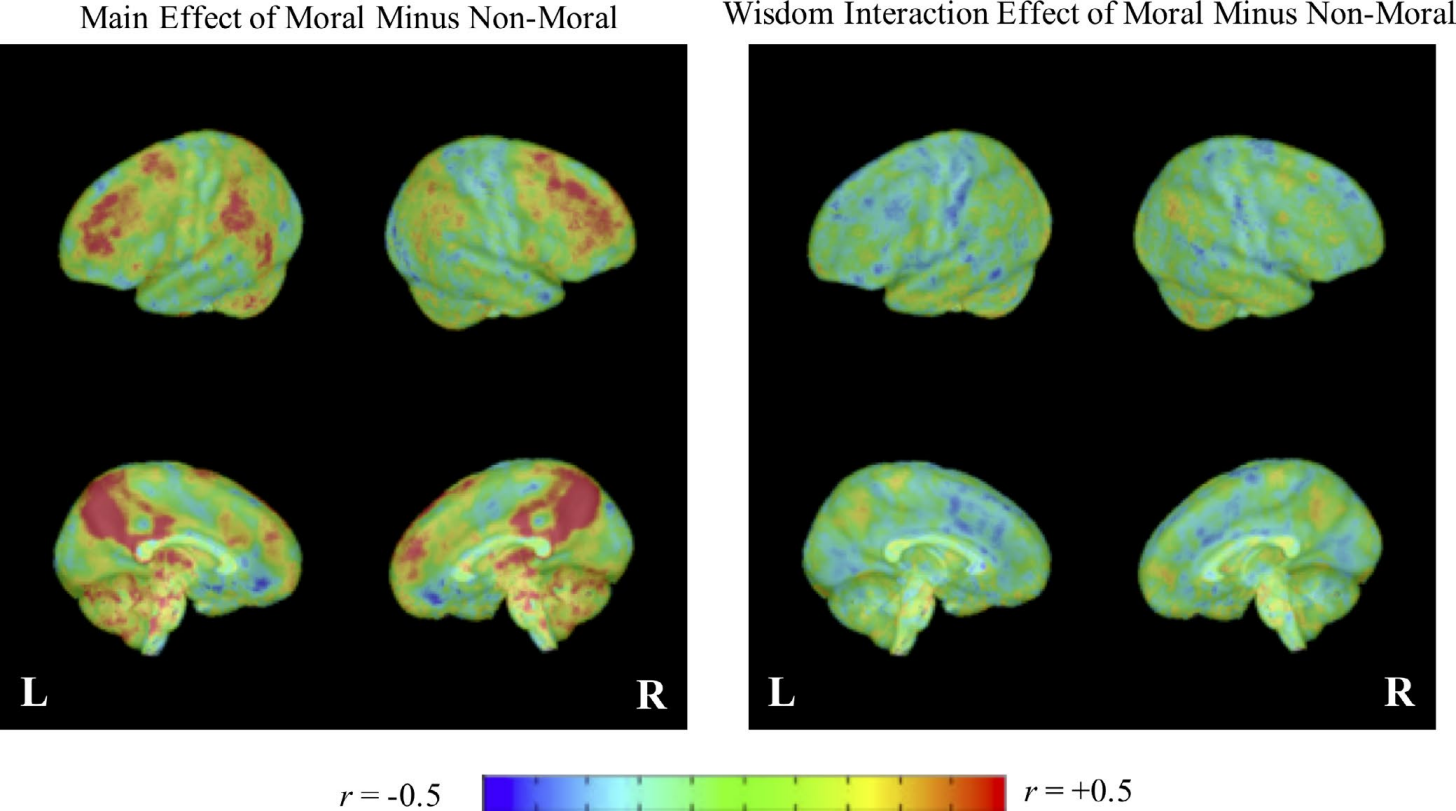
Whole brain standardized effect size display for the main and wisdom interaction effects of moral minus nonmoral conditions. Effect sizes are reported as correlations (using the *t*‐to‐*r* transformation). Redder/warmer colors indicate positive effects of strong magnitude and bluer/cooler colors indicate negative effects of strong magnitude. Greener/temperate colors indicate weak positive and negative effects

**Figure 5:**
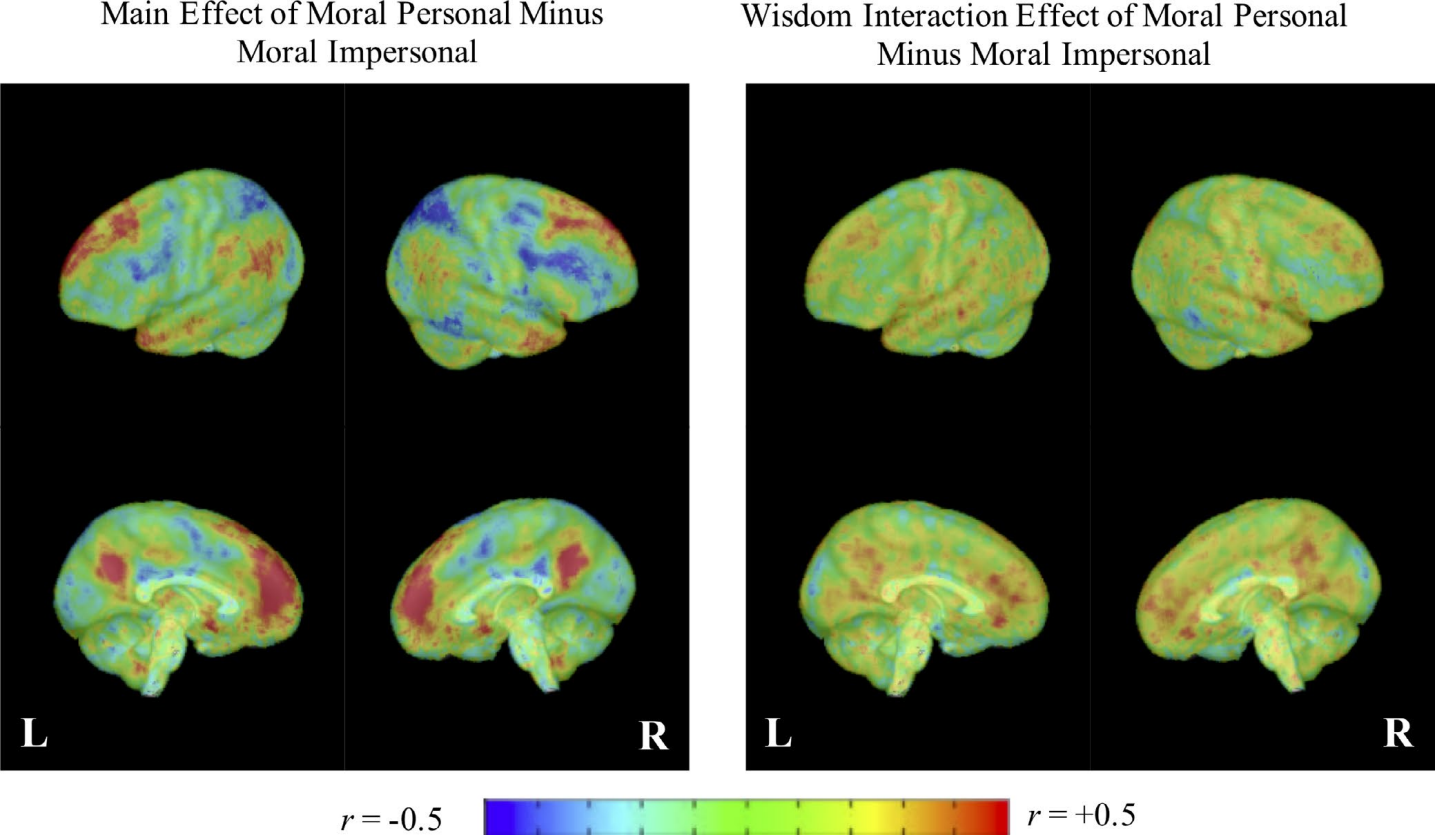
Whole brain standardized effect size display for the main and wisdom interaction effects of moral personal minus moral impersonal conditions. Effect sizes are reported as correlations (using the *t*‐to‐*r* transformation). Redder/warmer colors indicate positive effects of strong magnitude and bluer/cooler colors indicate negative effects of strong magnitude. Greener/temperate colors indicate weak positive and negative effects

To facilitate interpretation of inferential results as well as future studies of the neurophysiological correlates of wisdom, Figures 4 and 5 present standardized effect size maps (correlation coefficients) for the main and wisdom interaction effects of the contrasts between moral versus nonmoral and moral‐personal versus moral‐impersonal conditions, respectively, without thresholding for statistical significance. The figures largely support the ROI and whole‐brain voxel‐wise analyses, suggesting that higher SD‐WISE scores were associated with more negative moral versus nonmoral contrast effects (i.e., a relatively large proportion of negative [blue] vs. positive [red] voxels in the right panel of Figure 4) but more positive moral‐personal moral‐impersonal contrast effects (i.e., a relatively large proportion of positive [red] vs. negative [blue] voxels in the right panel of Figure 5).

## 4 CONCLUSIONS

The goal of this paper was to determine whether wisdom, as assessed by a psychometric scale, was associated with brain activation during a moral decision‐making task. We hypothesized that individual differences in wisdom would interact with moral condition in relation to brain activation, particularly in regions that are associated with reflective thinking (e.g., DMN).

The most consistent pattern of results, emerging across both ROI and whole‐brain voxel‐wise analyses, was the finding that participants who had higher SD‐WISE scores demonstrated relatively greater positive change in brain activation for the contrast of moral‐personal versus moral‐impersonal dilemmas—that is, wisdom was associated with enhanced contrast between the two types of moral dilemmas, with relatively greater activation in the moral‐personal condition. This pattern supported our hypotheses. The second pattern that emerged was relatively less positive change in the BOLD effect for the contrast of moral versus nonmoral dilemmas—that is, participants with higher SD‐WISE scores demonstrated a less distinct brain response when comparing moral and nonmoral conditions. This pattern did not support our hypothesis.

Why regions of the brain might be engaged differently by individuals with different levels of psychometrically measured wisdom is a question that cannot be answered by the results of this study. Although it is possible that wiser individuals engage different neurocognitive processes in the context of moral decision‐making than those with lower wisdom scores, we also cannot rule out the possibility that wisdom is associated with quantitatively, rather than qualitatively, different information processing (e.g., differences in efficiency). It is important to note, however, that response times were not associated with SD‐WISE scores. To the extent that response time is a valid indicator of the time participants spent deliberating, the results do not appear to be an artifact of the time on task.

Participants with higher SD‐WISE scores were more likely to choose utilitarian responses in the nonmoral condition in comparison to participants with lower SD‐WISE scores. Utilitarian responses, in this context, have a greater degree of “correctness” in comparison to moral decisions—that is, nonmoral utilitarian decisions maximize benefit without a moral cost. Figure 3 suggests that participants with higher SD‐WISE scores showed larger activation during the nonmoral condition in brain regions that are part of both the DMN and the cognitive control network (see Buckner, 2013). Similarly, the descriptive findings (Figure 4) suggest that greater wisdom is associated with less distinction in the neurophysiological response to moral versus nonmoral conditions. This could suggest greater mental effort overall, across multiple modes of thought, might have led to more utilitarian responding.

There were no significant associations between SD‐WISE scores and utilitarian responses made during the moral‐personal and moral‐impersonal conditions. And thus, there are no clear behavioral markers that might suggest why brain response in the contrast between these conditions was associated with level of wisdom. However, it is notable that the majority of brain regions showing wisdom associations for the contrast lie within the DMN (Raichle, 2015), a network that research suggests is more strongly engaged by moral‐personal decisions (Greene et al., 2001, 2004). The descriptive results (Figure 5) also suggest greater relative activation of brain regions within the salience network (Menon, 2015). Although only two of the regions within the salience network reached statistical significance, the results are nonetheless notable given that Chiong et al. (2013) suggested that the salience network is involved an alerting and switching during moral reasoning—that is, the salience network is theorized to be charged with identifying the personal nature of dilemmas, and then recruiting the DMN. It is possible that wisdom is associated with greater ability to recognize social and emotional cues, and thereby induce greater reflective thinking. Future studies that examine how connectivity between these regions is associated with wisdom might help support this suggestion.

Wisdom is commonly associated with emotional intelligence and theory of mind (Rakoczy, Wandt, Thomas, Nowak, & Kunzmann, 2018; Zacher, McKenna, & Rooney, 2013), domains that have also been shown to be related to the DMN (Mars et al., 2012). The common theme is that processes involved in wisdom, emotional intelligence, and theory of mind might reflect some aspect of imagined mental states, possibly related to the consequences of choices and their impact on one's own and others' feelings. For example, the significant ROI near the right IFGOr lies in an area that is associated with emotional response to embarrassing or rule violating behaviors (Berthoz, Armony, Blair, & Dolan, 2002) as well as inhibition (Aron, Robbins, & Poldrack, 2004, 2014), especially during reasoning and decision‐making (De Neys, Vartanian, & Goel, 2008; Goel & Dolan, 2003). The lateral PFC has also been shown to have an important role in theory of mind studies. Lesions in the lateral PFC affect the individuals' ability to stop focusing on their own experience and consider another person's state of mind (Samson, Apperly, & Humphreys, 2007; Samson, Apperly, Kathirgamanathan, & Humphreys, 2005). Without the lateral PFC, individuals might assume others share their viewpoint and cannot acknowledge alternative perspectives (Lieberman, 2007).

It is notable that a recent and growing literature supports the hypothesis that prosocial and moral behaviors are automatic rather than deliberative (Capraro, 2017; Capraro, Schulz, & Rand, 2019; Rand, 2016). Results of this literature seem to be in conflict with the assumption that emotional regulation and other cognitive control mechanisms facilitate wisdom. The observation that time pressure impacts moral decision‐making is an important consideration when interpreting results of this study. Indeed, the dynamics of the current experiment were restricted to a relatively short (time‐pressured) response window. It is possible that the influence of morality on wisdom, and thus the associated brain response, is influence by the deliberation time offered to participants. Future studies are needed to determine the impact of deliberation time on neural activity associated with wisdom and moral decision‐making.

Results of this study should be interpreted in light of several other certain limitations. First, the methods of this study were correlational in nature. Thus, causal links between wisdom and brain activation cannot be claimed. Second, individual differences in wisdom were quantified using a psychometric measure that, while previously shown to have good statistical properties (Thomas et al., 2017), is subject to biases, and represents a theorized construct rather than a known variable. Moreover, Grossmann (2017) and Grossmann et al. (2012) have demonstrated that wisdom is influenced by contextual features; a person's level of wisdom may depend, in part, on the context and manner in which that wisdom is assessed. Third, our sample was comprised primarily of well‐educated white individuals, and therefore, the results may not be generalizable to less educated or racial/ethnic minority groups. Finally, given the large amount of (within‐subject) data obtained using fMRI, we chose to apply statistical methods that guard against inaccurate claims of statistical association. This might have caused us to miss some meaningful associations between wisdom and brain activation, if those associations were in the small to medium effect size range.

The current findings establish a basis for the association between neurophysiological processes and wisdom that must be replicated and followed by carefully controlled experimental studies. Direct manipulation of brain regions, thought to be involved in wisdom (e.g., possibly using transcranial magnetic stimulation), would provide stronger tests of the theory. Studies suggest that traits such as empathy and compassion can be altered through training within the context of a randomized intervention, and that these changes are associated with alternative brain activation (Klimecki, Leiberg, Lamm, & Singer, 2013). Whether such changes are possible in the area of wisdom is yet unknown, but warrants further investigation given our growing understanding of the importance of wisdom.

In summary, we have provided preliminary support for a neurophysiological basis of wisdom. In particular, level of wisdom, as measured with a psychometrically validated instrument, appears to moderate the effects of moral reasoning on brain response. Participants with higher wisdom scores demonstrated relatively greater engagement of brain regions within the DMN for moral‐personal conditions, which, we suggest, might serve to enhance the ability to recognize and process social and emotional cues that are relevant to decision‐making.

## DECLARATION OF INTEREST

None to declare.

## ROLE OF THE FUNDING SOURCE

The funding organizations had no role in the design and conduct of the study; collection, management, analysis, and interpretation of the data; and preparation, review, or approval of the manuscript; and decision to submit the manuscript for publication.

## Data Availability

Data collected for this study are not part of a public data repository.
