# Supplementary material for: Individual differences in level of wisdom are associated with brain activation during a moral decision‐making task
Source: Brain Behav. 2019 May 1;9(6):e01302. doi: 10.1002/brb3.1302 (PMC6577614; doi:10.1002/brb3.1302)
Supplement: Supplementary file 1 [file BRB3-9-e01302-s001.docx]

******

***Supplemental Figure 1*.** **Preprocessing**

Local scripts as well as software from AFNI and FSL were used to process the structural and functional images. Beginning with the anatomical images, we first removed non-brain tissue. In cases where the automated routines performed less than optimally, adjustments were performed manually. Anatomical images were registered into Talairach space using the ICBM-452 brain template. Next, the multiband functional images were reconstructed and distortions due to inhomogeneities in the magnetic field were corrected. Scanner artifacts were removed and then we co-registered the functional images and then aligned them to the structural images. After identifying outliers, we then then blurred the images.
